# Inflight fiber printing toward array and 3D optoelectronic and sensing architectures

**DOI:** 10.1126/sciadv.aba0931

**Published:** 2020-09-30

**Authors:** Wenyu Wang, Karim Ouaras, Alexandra L. Rutz, Xia Li, Magda Gerigk, Tobias E. Naegele, George G. Malliaras, Yan Yan Shery Huang

**Affiliations:** 1 Department of Engineering, University of Cambridge, Cambridge CB2 1PZ, UK.; 2 The Nanoscience Centre, University of Cambridge, Cambridge CB3 0FF, UK.

## Abstract

Scalability and device integration have been prevailing issues limiting our ability in harnessing the potential of small-diameter conducting fibers. We report inflight fiber printing (iFP), a one-step process that integrates conducting fiber production and fiber-to-circuit connection. Inorganic (silver) or organic {PEDOT:PSS [poly(3,4-ethylenedioxythiophene) polystyrene sulfonate]} fibers with 1- to 3-μm diameters are fabricated, with the fiber arrays exhibiting more than 95% transmittance (350 to 750 nm). The high surface area–to–volume ratio, permissiveness, and transparency of the fiber arrays were exploited to construct sensing and optoelectronic architectures. We show the PEDOT:PSS fibers as a cell-interfaced impedimetric sensor, a three-dimensional (3D) moisture flow sensor, and noncontact, wearable/portable respiratory sensors. The capability to design suspended fibers, networks of homo cross-junctions and hetero cross-junctions, and coupling iFP fibers with 3D-printed parts paves the way to additive manufacturing of fiber-based 3D devices with multilatitude functions and superior spatiotemporal resolution, beyond conventional film-based device architectures.

## Teaser

From capturing your breath to “floating” 3D electronics, inflight printing of tiny, invisible conducting fibers does the magic.

## INTRODUCTION

Small-diameter conducting fibers have unique morphological, mechanical, and optical properties such as high aspect ratio, low bending stiffness, directionality, and transparency that set them apart from other classes of conducting, film-based micro/nano structures (*1*–*3*). Orderly assembling of thin conducting fibers into an array or three-dimensional (3D) structures upscales their functional performance for device coupling. Developing new strategies to control rapid synthesis, patterning, and integration of these conducting elements into a device architecture could mark an important step in enabling new device functions and electronic designs (*4*, *5*). To date, conducting micro/nanoscaled fibers have been produced and assembled in a number of ways, from transferring of chemically grown nanofibers/wires (*6*, *7*), writing electrohydrodynamically deposited lines (*8*, *9*), to drawing ultralong fibers (*10*, *11*), wet spinning of fibers (*12*–*14*), and 2D/3D direct printing (*15*–*18*). These unique conducting structures have enabled a number of applications, including transparent and flexible electrodes (*19*, *20*), conducting yarns (*21*), tissue engineering scaffolds (*22*), and bioelectronic devices (*23*).

Despite these developments, existing fabrication techniques do not readily allow the assembly of fiber architectures leading to device functions that exploit combinations of the unique fiber characteristics: directionality, high surface area–to–volume ratio, and permissiveness, along with transparency and conductivity. Given their small-diameter (micro/nanoscaled) and high aspect ratio (>100), manipulation of thin fibers at the single-fiber level could be intricate and slow. Bonding and connecting the fibers with minimized contact resistance presents a major challenge for circuitry creation. As a result, most existing applications of fiber-based transparent conductors are substrate based (*17*, *24*). The underlying substrates can dominate the optomechanical properties of the transparent conductor in the final device setting; further, the permissive, high surface area–to–volume ratio qualities of a fiber array are completely lost. In addition, from fiber synthesis to device integration, a multistep procedure or postsynthesis fiber treatments are often required to obtain adequate on-circuit, fiber bond-to-bond conductivity (*10*). This, in turn, restricts the materials library of conducting fibers that can be used.

Here, we develop inflight fiber printing (iFP): a one-step process working at sub-100°C, which creates and in situ bonds thin conducting fiber arrays, either suspended or on a surface, without needing any postprocessing. By optimizing the fiber sizing, we show iFP as a versatile technique for rapid on-circuit creation of small-diameter (~1- to 3 μm thick) conducting fibers of vastly dissimilar natures. As exemplified cases, we produce inorganic, metallic silver fibers from solution-based reactive synthesis and organic conducting PEDOT:PSS [poly(3,4-ethylenedioxythiophene) polystyrene sulfonate] fibers. PEDOT:PSS fibers are of special interest for biological applications considering the difficulty in producing PEDOT:PSS fibers with diameters below the typical cell size (a few micrometers), because the low-viscosity properties of the PEDOT:PSS solutions, which are suited for thin-film production supported by a substrate, hamper the micro/nano fiber production. With iFP’s ability to fabricate fibers on circuit, we exploited the distinctive and versatile functional advantages of the fiber array, namely, permissiveness, directionality, and high surface area–to–volume ratio, in applications covering flow-permissive moisture sensing, noncontact respiratory moisture sensing, and bioimpedimetric sensing. We then explore novel circuitry architecture afforded by the iFP process, presenting the concept of 3D “floating electronic architectures,” and merging organic and inorganic fiber materials in the same transparent conducting network.

## RESULTS

### Inflight fiber printing

**Fig. 1:**
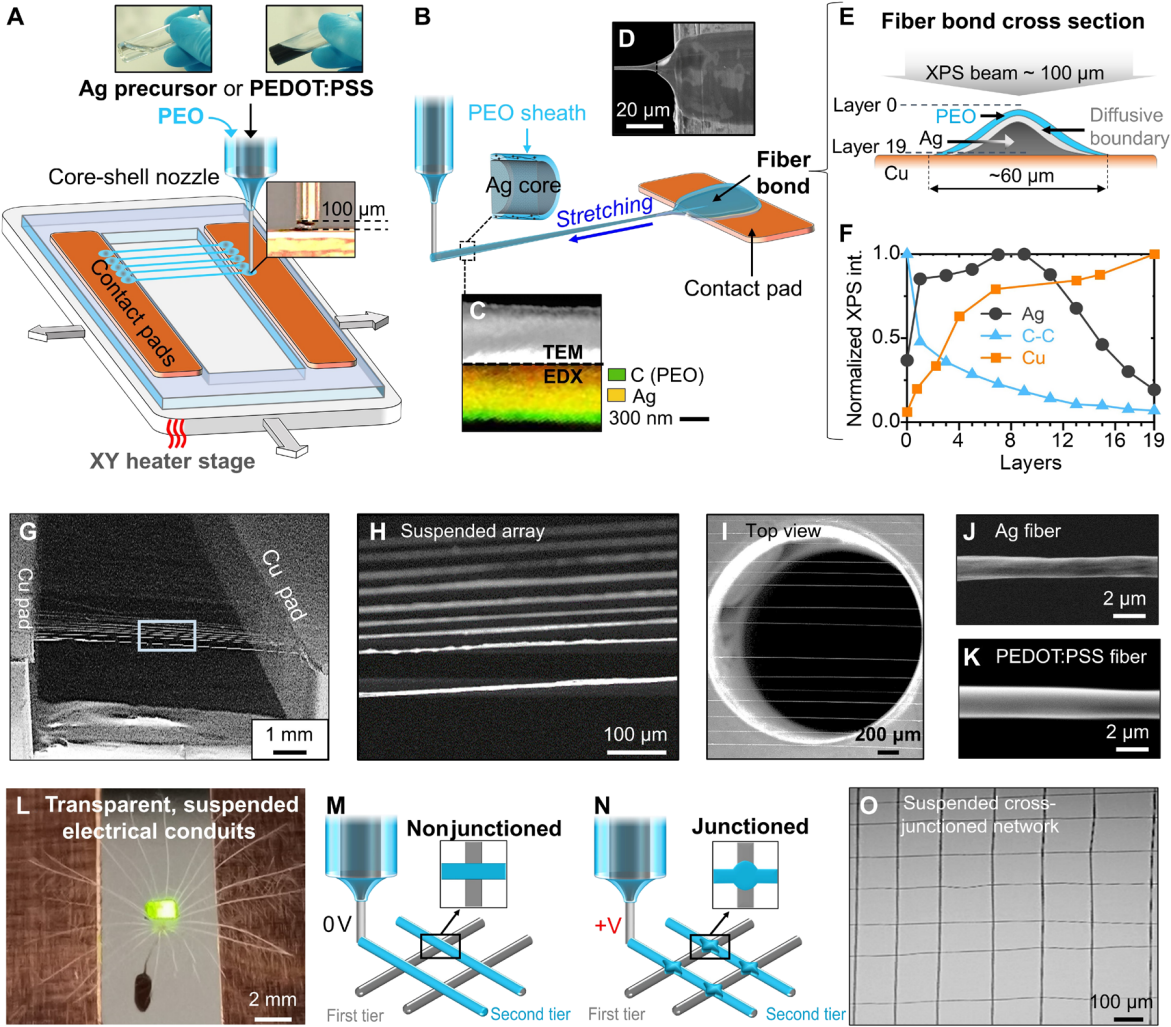
iFP fabrication of suspended fiber structures with in situ bonding. (**A**) Schematic of iFP process for Ag and PEDOT:PSS fibers. (**B**) Schematic showing a close view of the initiation of the iFP fibers. (**C**) TEM and EDX of a single Ag fiber. (**D**) Scanning electron microscopy (SEM) image showing fiber bond from the top view. (**E**) Cross-sectional schematic of fiber bond. (**F**) XPS depth profiling on the Ag fiber bond. (**G** and **I**) SEM images of typical suspended, aligned fiber array. (**J** and **K**) SEM images showing individual Ag and PEDOT:PSS fibers. (**L**) Image of a powered LED lamp and a dandelion seed on top of a suspended PEDOT:PSS fiber array, with the seed passing through the fiber array (Photo credit: Wenyu Wang, University of Cambridge). (**M** and **N**) Schematics of nonjunctioned and junctioned fiber grid structures. (**O**) Optical image of a suspended iFP fiber network.

The iFP process is schematically shown in Fig. 1A. Silver (Ag) and PEDOT:PSS are used as the fiber materials, with Ag being a widely used conducting metal (*25*) and PEDOT:PSS a biocompatible conducting polymer with applications ranging from textile (*26*), printed electronics (*27*), to biointerfacing structures (*28*). To reflect the universality of the process, we derive fibers from readily available aqueous solutions of low viscosity (~10 to 60 mPa·s). It is to note that higher viscosities aqueous solutions, i.e., with viscosity of ~10^4^ mPa.s [such as 22% (w/w) gelatin solution] can also be processed by iFP (fig. S1A). For Ag, we demonstrate reactive synthesis of fibers from a modified Tollens’ solution containing an Ag salt and a reducer (*29*). This differs from commonly used metal nanoparticle-based conducting inks in fiber production processes that are generally prepared by mixing metal nanoparticles with polymers. For PEDOT:PSS fibers, a commercial aqueous colloidal solution is used (see Materials and Methods).

The iFP process uses a core-shell nozzle, delivering an aqueous sizing agent in the sheath [polyethylene oxide (PEO); (weight-average molecular weight) *M*_w_ = 4 million Da, 2% (w/w) in deionized (DI) water] to ensure the core continuity and to prevent interface instability. Sub-100°C heating is applied to rapidly evaporate the solvent and to simultaneously activate the Ag precursor in the case of Ag fiber production. The iFP technique does not require preprocessing or additives to tune the viscosity of the core solution, as the fiber structure can be transiently maintained by the sizing polymer in the sheath during fiber synthesis. This differs from usual printing techniques in which the viscosity of inks needs to be optimized beforehand (*30*); hence, our technique supports a variety of functional materials to be directly used in their original forms as the fluid core, such as PEDOT:PSS and gelatin solutions and metallic precursor solutions (e.g., silver and copper precursors).

At the beginning of fiber synthesis, the tip of the core-shell nozzle is positioned at ~100 μm above the surface of the heated contact pad, allowing the pendant drop from the nozzle to adhere to the contact pad. Repeated stage translations lead to parallel fibers bridging the suspension gap between two contact pads (Fig. 1A). A nozzle to contact pad separation distance smaller than 100 μm would cause nozzle clogging because the heated contact pad would instantly solidify the solution in the as-extruded core-shell drop, while a larger distance could cause unsuccessful fiber initiation (fig. S2A). A close view of the fiber formation is schematically shown in Fig. 1B, with the dynamic process demonstrated in movie S1. Initially, the pendant drop on the contact pad becomes a fiber bond, and then the stage translation leads to capillary breakup of the droplet to initiate a fiber. The fiber is produced by both the mechanical stretching and the heating. Mechanical stretching thins the fibers, while the heating accelerates solvent evaporation. The core solution will eventually solidify into the conducting phase for both Ag and PEDOT:PSS fibers. Subsequently, the fiber reaches the opposite contact pad and forms another fiber bond, bridging the two electrodes. TEM (transmission electron microscopy) micrograph and EDX (energy-dispersive x-ray) mapping of an Ag fiber is shown in Fig. 1C. The TEM micrograph shows a continuous core of silver (appears white in the image because of stronger diffraction from the heavier compounds), surrounded by a thin sheath of PEO (appears translucent because of lighter organic compounds). The TEM image also reflects the continuity of the core in the longitudinal direction. Chemical mapping by EDX of Ag-PEO fiber on the bottom side of Fig. 1C further confirms that the core is dominated by silver species, while the sheath is dominated by carbon species that represent the PEO polymer.

The fiber bonds (Fig. 1D) serve the electrical connection between the fiber and the electrical contact pads. The direct connection between the electrical pad and the conducting phase (e.g., Ag) of the fiber bond, with minimal interference of the PEO sizing polymer, is evidenced by x-ray photoelectron spectroscopy (XPS) depth profiling (Fig. 1, E and F). An ion gun is used to etch the fiber bond and Cu contact pad junction area in the depth direction, and XPS high-resolution spectra of silver 3d and carbon 1s are acquired at each etching layer. At all depth layers, main peaks at 368.3 and 374.3 eV correspond to Ag 3d_5/2_ and Ag 3d_5/3_ binding energies, and the main peak at 284.7 eV corresponds to C 1s (C-O) binding energy (fig. S3, A and B) (*19*) Because of the complexity of the materials at the fiber bond area, it is not straightforward to estimate the precise etching distance from the etching time and energy. However, given that the electrical contact pad is made of copper, the Cu_2p_ intensity values at each layer were used to provide an estimation of the distance between the current etching layer and the copper electrode (fig. S3C). We should mention that the ion beam diameter (~100 μm wide) is much larger than the fiber bond (~60 μm wide), explaining why Cu_2p_ peak is detected in all layers during the XPS depth analysis. At layer 0, the top surface of the fiber bond, the silver intensity is weak while the carbon intensity is strong, meaning that the surface of the fiber bond is mainly covered by PEO. When etching down the fiber bond, the silver intensity starts to increase and the carbon intensity keeps decreasing, indicating that the silver is mainly covered underneath the PEO. The copper intensity reaches its maximal value at around layer 19, meaning that the surface of the copper electrode contact pad has been reached. The fact that the carbon content decreases monotonically from the top of the fiber bond to the surface of the copper electrode pad suggests that there is a negligible polymer-rich barrier between the silver phase and the copper electrode contact pad. The XPS depth profiles support that silver is directly in contact with the copper electrode contact pad, forming a continuous electrical pathway from the electrical contact pads to the fibers. To assess the characteristics of PEODT:PSS fiber bonding, a transmission line method experiment is conducted. As shown in fig. S4A, the resistances of PEDOT:PSS fibers with different lengths have been measured, and the linear extrapolation shows that the contact resistance (when fiber length is 0 mm) is negligible (i.e., an electrical conducting fiber bond is achieved).

Aided by the sizing polymer to form the sheath of the iFP fibers, the core phase of the fibers only consists of substances of the silver precursor solution, without an interpenetrating polymer that binds and separates the synthesized silver particles. Therefore, the critical temperature required to obtain a connected metallic core in iFP system is expected to be controlled by the initiation temperature of silver reaction and solvent evaporation (~ 60° to 80°C), rather than by the calcination temperature (>250°C) of the polymer binder as reported in previous studies (*11*, *19*). Using iFP, we demonstrate that a one-step process at sub-100°C is sufficient to obtain adequate fiber conductivity (see next section), in comparison with usual fiber production techniques that require postprocessing such as sintering (*11*, *19*). Moreover, as iFP proceeds at a temperature lower than 100°C, this would create opportunities for the direct integration of both PEDOT:PSS and Ag fibers into a wider range of materials and structures. In the special cases of printing short suspended (<2 mm) or on-substrate PEDOT:PSS fibers, iFP could operate entirely at room temperatures. The iFP process is foreseen to be most suitable for applications that require reduced temperatures and mild fabrication environments, such as for incorporating biologic factors and thermosensitive materials.

### Morphological and opto-electro-mechanical properties of iFP fibers

Typical micrographs of aligned, suspended conducting fiber arrays are shown in Fig. 1 (G to I). Such an array of around 100 fibers can be printed within 1 min, thanks to the high printing speed (600 mm/s). This makes iFP an efficient fiber printing process. The iFP allows parallel fiber array printing with the smallest fiber-to-fiber separation spacing of ~75 μm, which is mainly limited by the size of the fiber bond (~ 60 μm in width). When the fiber spacing is smaller than ~75 μm, the fiber bond, which is created by the pendent drop, would overlap; thus, the pendent drop would interfere with the previously printed fibers (fig. S2B). However, the fiber spacing could be tuned freely beyond 75 μm, as exemplified in fig. S2B. The upper spanning distance of the suspended fibers is mainly controlled by the molecular weight of the sizing polymer solution (i.e., the PEO sheath), if one intends to minimize the shell polymer concentration to maximize the functional core volume (see further explanation in fig. S2C). With the 2% (w/w) 4 million Da PEO sizing solution chosen in our case, suspended fibers with a spanning distance up to 10 mm can have their diameters altered between ~700 nm and ~3.2 μm. This is achieved by changing the overall flow rates from 20 to 120 μl/hour while keeping the shell-to-core flow rate ratio at 2:1. A summary and discussion of how fiber diameter, suspension morphology, and conductivity could be affected by the shell-to-core flow rates is shown in fig. S5. To demonstrate fiber-based sensing and device applications, we adapt core and shell flow rates of 25 and 50 μl/hour, respectively, in iFP, which result in a fiber diameter of 2 ± 0.7 μm for both Ag and PEDOT:PSS fibers. Scanning electron microscopy (SEM) micrographs (Fig. 1, J and K) show that these fibers exhibit a circular fiber geometry.

A photo of the resulted suspended fiber array, on which a light-emitting diode (LED) lamp is suspended with a dandelion seed, is shown in Fig. 1L. The ~1- to 3-μm diameter of iFP fibers makes the array appear to be visually transparent, even comparing with the fine hairs of the dandelion. Because of the substrate-free nature of the fiber array, the hairs and stem of the seed pass freely through the fiber array while being supported, showing the permissiveness of iFP fiber array. This suspended electrical conduit illustrates both the transparency and conductivity of the fiber array. Alternatively, the fiber arrays can be combined to form a network structure, in which the fiber materials can be varied in different tiers of the same grid. In iFP, the freshly printed fibers exhibit a transient semiliquid state, which can be exploited for controlling the crossing points in the grid, to be structured as a nonjunctioned or junctioned network (Fig. 1, M and N, and see further discussion in a later section). Such fiber network pattern is illustrated by the optical image of suspended cross-junctioned fiber network shown in Fig. 1O. In addition, by applying a small voltage at the nozzle (~150 V), fibers can be bound onto different substrate surfaces of either conducting or insulating nature (fig. S1B), demonstrating flexible switch between “spanning” and “on-substrate” printing.

**Fig. 2:**
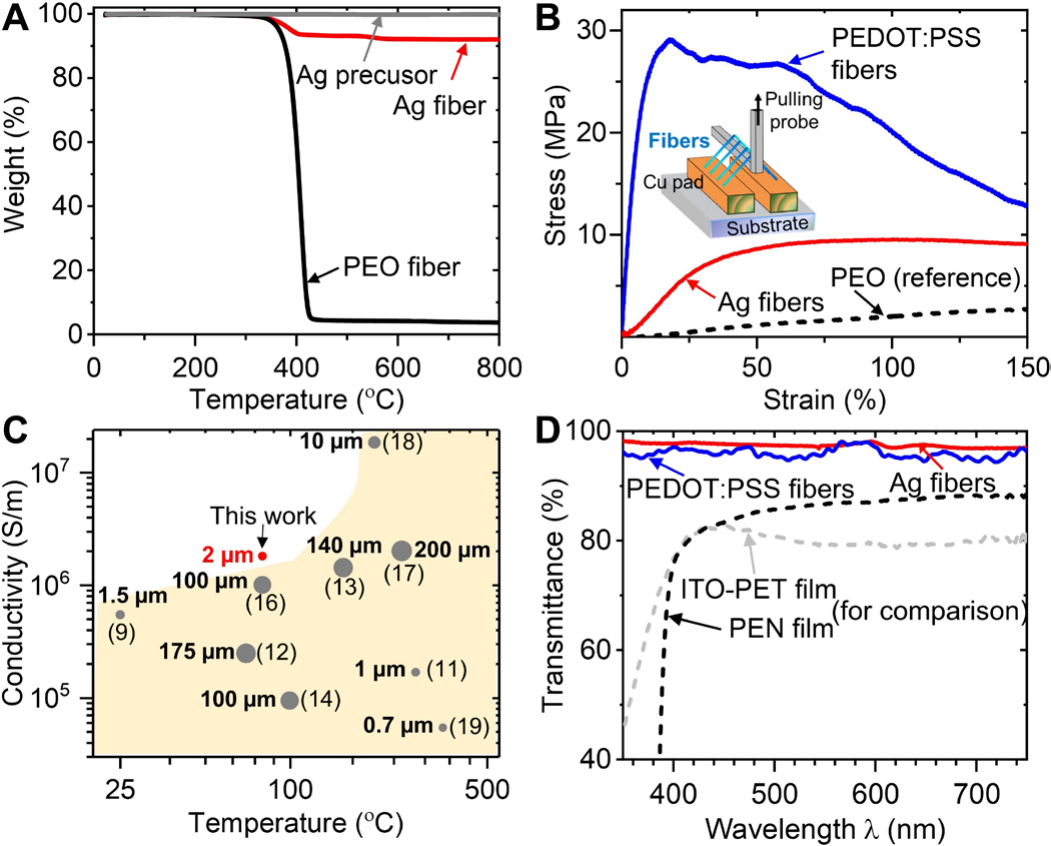
Opto-electro-mechanical characteristics of iFP fiber array. (**A**) TGA of Ag fibers, with pure PEO fibers and solidified Ag precursor as references. (**B**) Stress-strain curves of Ag, PEDOT:PSS, and pure PEO fibers. (**C**) Summary of Ag fiber performance comparing processing temperature, conductivity, and fiber diameter with literature (the ● symbol size is proportional to the fiber diameter or feature width). (**D**) Transmittance of Ag fiber array, PEDOT:PSS fiber array, and PEN (polyethylene naphthalate) and ITO/PET films versus wavelength.

Further investigations of the Ag fiber composition using TGA (thermogravimetric analysis) have been performed to estimate the mass fraction of silver content (Fig. 2A). The weight loss of around 7% from 300° to 400°C can be attributed to the thermal decomposition of PEO, which is verified by using pure PEO fibers as reference. Above 400°C, there is no evident weight loss because of the high thermal stability of silver content in the solidified Ag precursor. This indicates that Ag-PEO fibers have more than 90% (w/w) of silver content. X-ray diffraction results of the Ag-PEO fibers (fig. S3E) support the presence of normal silver crystal phases associated with elemental silver. The crystallinity of the silver in the Ag fibers is ~51.8% (w/w) from the XRD spectroscopy, and the average particle size is determined as ~33.9 nm (table S1A). In addition, cross-sectional SEM images in fig. S3F also show that the closely packed silver particles inside the Ag fiber are of tens of nanometers. The overall crystallinity and crystalline size of the iFP Ag fibers compare similarly with literature values of solution processed silver line/fiber features (crystallinity 25 to 60% and crystalline size 20 to 80 nm) (*31*, *32*). However, high temperatures (i.e., > 200°C) and postprocessing (i.e., sintering) are usually needed in those approaches; in comparison, iFP operates at sub-90°C in a single step to produce the silver fibers. In other words, when printing fibers at sub-100°C, most of the silver precursors have been converted to elemental silver in the fiber core.

To evaluate the mechanical properties of the suspended fiber array, tensile test is performed on suspended Ag, PEDOT:PSS, and pure PEO fibers (Fig. 2B). Both Ag and PEDOT:PSS fibers exhibit Young’s modulus and tensile strength lower than their pure core phase counterparts. This is as expected since the PEO sizer has a low Young’s modulus (~2 MPa) and yield strength (~5 MPa), and the overall mechanical properties of the fibers are determined by both the PEO sizer and the core materials. As observed in Fig. 2B, for Ag fibers, the yield strength and Young’s modulus in the elastic regime are *E* ~ 10 MPa and σ_y_ ~ 15 MPa, respectively. Upon stretching, the compacted nano silver particles inside the Ag fibers would disconnect more easily compared to polycrystalline silver, but the polymer shell could retain the bulk fiber integrity. The TEM images from fig. S3G show the silver core rupture inside the fiber sheath. However, the high viscoplasticity of the PEO sheath enhances the strain to mechanical failure of the fiber (failure strain >100%). It is noteworthy that these values are similar to the mechanical properties of human skin (*33*), potentially benefiting applications as direct fiber-based skin electronics. The yield strength of PEDOT:PSS fiber is ~30 MPa, and the Young’s modulus is ~150 MPa. In contrast to nanoparticle-based conducting inks where metal nanoparticles are mixed with polymers, the iFP offers a unique core-shell fiber structure, separating the polymer sheath from the conducting phase in the fiber. This ensures the conducting materials to form a continuous fiber core, achieving high conductivity and unique mechanical properties.

In the case of Ag fibers, ~10^6^ S/m bond-to-bond fiber conductivity is obtained under a processing temperature of 80°C. Figure 2C shows that the conductivity and fabrication temperature of Ag fibers obtained by iFP compare favorably with those of existing literature. In particular, our data directly reflect the bond-to-bond fiber conductivity, having encompassed the contact resistance normally incurred in a device setting. With regard to the PEDOT:PSS fibers, a bond-to-bond conductivity of about 400 S/m and an upper spanning distance of 10 mm are obtained at ~65°C and shell-to-core flow rate ratio at 2:1. By decreasing the shell-to-core flow rate ratio (thus increasing the PEDOT:PSS solution volume fraction) during printing, the fiber conductivity can increase substantially, at the expense of reduced fiber suspension distance. The highest conductivity (~7000 S/m) can be achieved at a shell-to-core flow rate ratio of ~0.67, with an upper fiber suspension distance of ~5 mm (fig. S4B). Nevertheless, the 7000 S/m fiber conductivity compares favorably with the other reported PEDOT:PSS fiber systems (*34*, *35*). Overall, the iFP technique produces fibers of good conductivity at low processing temperatures (<100°C). Since iFP can produce thin spanning fibers, the “substrate-free” conducting fiber arrays even have better transparency compared with a standard transparent conductor film, i.e., ITO/PET (indium tin oxide–coated polyethylene terephthalate) with similar directional conductance (Fig. 2D). At a fiber density of 20 fibers/mm, the suspended Ag fiber array exhibits more than 95% transmittance in the 350- to 700-nm regime. The benefits of enhanced light transmission is especially obvious for the near-ultraviolet (UV) (below 400 nm) region, where the transmittance of the commonly used transparent films decreases sharply (see fig. S6A for absorption coefficient versus wavelength). The optoelectrical property of the suspended iFP fiber array makes them suitable for fulfilling applications where UV spectral region is involved.

### Flow-permissive moisture sensor

Along with the favorable optoelectrical characteristics, the free-standing feature of the iFP fiber array provides a unique advantage of being permissive to moisture flow. As a theoretical estimation, the small iFP fiber size (~2 μm in diameter) should exhibit a high surface area–to–volume ratio (~2 × 10^6^ per unit length in international systems unit), which is 50 times higher than a typical strand of human hair (~100 μm in diameter) at the same length for a single fiber. Such high surface area–to–volume ratio and the free-standing nature of the iFP fibers are expected to allow rapid mist absorption and dissipation with the ambient humidity (which act as the reservoir).

**Fig. 3:**
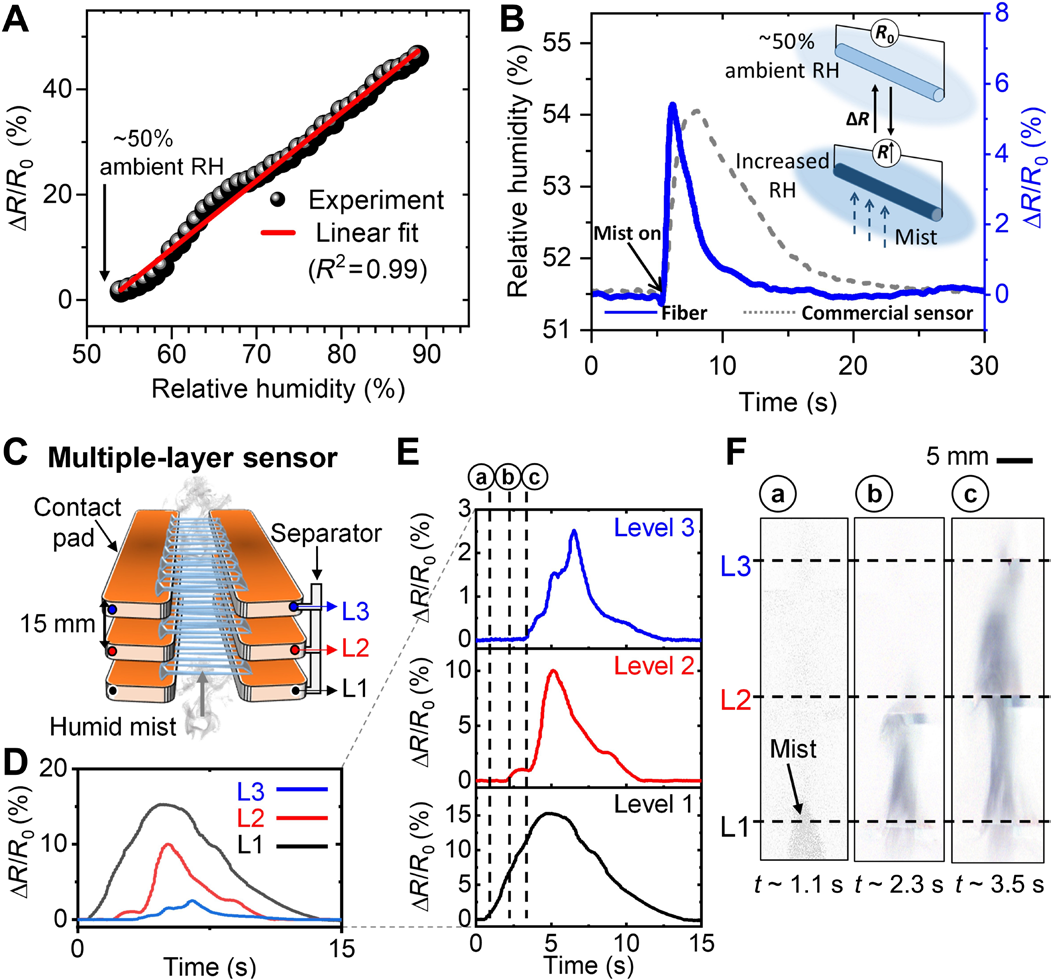
Flow-permissive moisture sensor. (**A**) iFP fiber array resistance plot showing the linear response of resistance as a function of relative humidity above room humidity (RH). (**B**) Commercial humidity sensor versus iFP PEDOT:PSS sensor signal in response to a moisture pulse. (**C**) Schematic of the multilayer sensor with three tiers (L_1_ to L_3_), with the corresponding time-resolved normalized fiber resistance change (Δ*R*/*R*_0_) in (**D** and **E**) and processed images of mist rising up from L_1_ to L_3_ in (**F**).

In this context, we have exploited the PEDOT:PSS fiber array as a sensing electrode for detecting spatiotemporal dynamics of moisture flows. In comparison with conventional fiber-based moisture-sensing materials (*36*), the iFP PEDOT:PSS fiber relies on measuring the end-to-end fiber resistance evolution, thus allowing tailored signal readout dependent on the fiber array organizations. Figure 3A shows that the normalized fiber resistance change (Δ*R*/*R*_0_) increases approximated linearly with respect to the relative humidity from 55 to 90% at room temperature (as calibrated with a digital hygrometer, Onecall Farnell). Compared to a commercial humidity sensor (HIH-5031-001, Honeywell), the iFP fibers demonstrate a faster response upon the impingement of a pulse humid mist (~1-s time resolution versus ~4 s) as shown in Fig. 3B. Since the fiber resistance values always return to the baseline value [i.e., equilibrating with room relative humidity (RH) level of ~50%] after removal of the mist, the influence of moisture absorption on the fiber resistivity is a reversible process. With this capability, the dynamics of moisture flow can be monitored by using a stacked fiber array in a 3D configuration where the moisture flow can freely pass through the fiber arrays (Fig. 3C), and the spatiotemporal resolution of detection is defined by the distance between two adjacent tiers of fiber arrays. Typically, a pulse mist applied from the bottom rises to encounter with level 1 to level 3 sensing tiers sequentially. The Δ*R*/*R*_0_ in each fiber level is recorded as exampled in Fig. 3D. Because of height difference of the fiber level, the Δ*R*/*R*_0_ is recorded at ~18% (SD = 2.5%) for the level 1 fiber array, ~10% (SD = 1.6%) for level 2, and ~5% (SD = 0.7%) for level 3 (fig. S6B). Simultaneously, imaging of the flow dynamics can be performed considering the transparent characteristic of the multilevel fiber-based electrodes. The time-resolved fiber resistance at each sensing tier is represented in Fig. 3E. The onset of resistance change has a time delay between subsequent sensing tiers of about ~1 s, corresponding well to the flow dynamics captured with a high-speed camera (Fig. 3F). This confirms the responsiveness and sensitivity of the spatiotemporal dynamic sensing. We should mention that the contrast of the mist in the processed images is due to in- and out-camera focus differences and does not reflect the water concentration distribution.

### Noncontact, wearable/portable respiratory moisture sensors

**Fig. 4:**
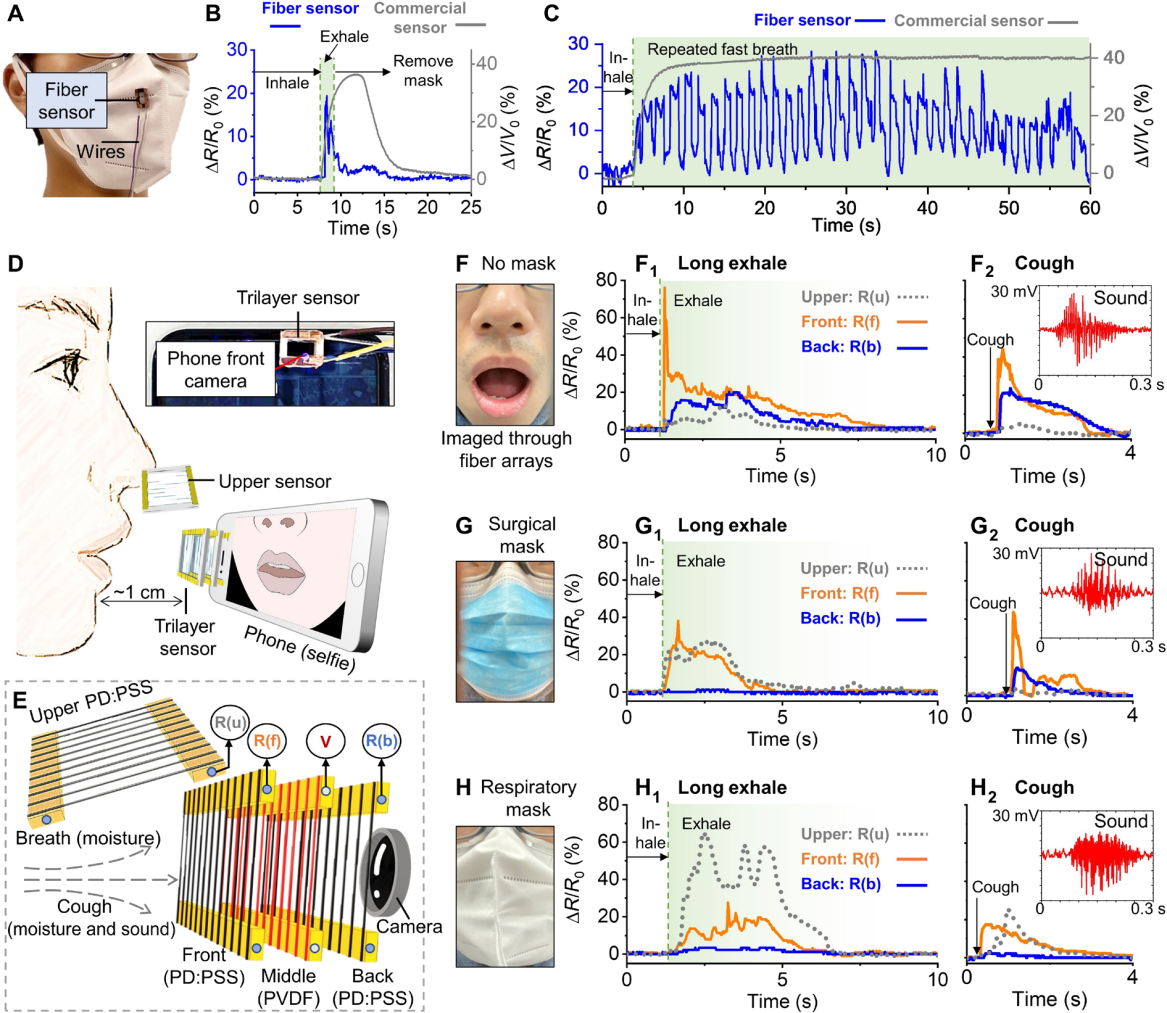
Fiber-based, noncontact respiratory moisture sensors. (**A**) Schematic showing a single-layer fiber wearable breath sensor attached to a disposable mask. (**B**) Commercial humidity sensor versus the iFP PEDOT:PSS fiber array in response to a single breath and continuous fast breathing (**C**). (**D**) Schematic showing a portable trilayer fiber sensor attached onto the front camera of a cell phone and a single-layer fiber sensor positioned above the nose. (**E**) Zoomed-in schematic of the fiber layer arrangement of the trilayer fiber sensor. PVDF, polyvinylidene difluoride. (**F**) Photo of the mouth area in the “no face covering.” (**F**_**1**_) Normalized fiber resistance change (Δ*R*/*R*_0_) of long exhale breath and cough (**F**_**2**_), with the inset showing cough sound detected by the middle piezoelectric fiber layer. (**G**) Photo showing a surgical mask wearer, with fiber sensor recordings during long exhale (**G**_**1**_) and cough (**G**_**2**_). (**H**) Photo of a respiratory mask wearer, with fiber sensor recordings during long exhale (**H**_**1**_) and cough (**H**_**2**_). It is to note that photos of (F), (G), and (H) were all captured by the phone camera through the trilayer fiber sensor (Photo credit: Wenyu Wang, University of Cambridge).

In the global outbreak of acute respiratory disease, such as coronaviruses, influenza, and rhinovirus, guidance on the effects of face mask wearing could help mitigate the risk of disease transmission (*37*). On the basis of the fact that human breath contains highly humidified gas (89 to 97% RH) (*38*), we fabricated two configurations of high-sensitivity, low-cost, and wearable/portable respiratory moisture sensors with the iFP PEDOT:PSS fiber arrays (Fig. 4). In the first fiber sensor configuration, a single-layer PEDOT:PSS fiber array is printed on a 3D-printed plastic frame. This fiber sensor can be readily attached to the exterior of a disposable mask as a wearable, noncontact, and noninvasive sensor for respiration rate monitoring (Fig. 4A and movie S2). As shown in Fig. 4B, the iFP fiber respiratory moisture sensor shows superior responsiveness compared to a commercial humidity sensor (HIH-5031-001, Honeywell). The resistance of the fiber array returns to the baseline level in less than 3 s after a normal breathing (versus ~10 s associated with the commercial sensor). This effect is particularly pronounced for applications such as fast breathing detection (~1.2 s per breath cycle, simulating fast breath under intensive exercise or shortness of breath), where the commercial sensor fails to distinguish individual breathing events (Fig. 4C).

The second fiber sensor configuration is a trilayer 3D sensor, which consists of PEDOT:PSS fiber arrays as the front and back layers, sandwiching a suspended poly(vinylidene fluoride-*co*-trifluoroethylene) [P(VDF-TrFE)] nanofiber midlayer (Fig. 4, D and E). The midfiber layer of P(VDF-TrFE) fibers, which is fabricated separately by dynamic near-field electrospinning (*39*), is used to detect sound by acoustically driven piezoelectricity. As the entire 3D sensor is transparent and air permissive, this sensor can be attached to a phone camera for simultaneous collection of multilatitude information, i.e., image, sound, and local spatial variation in breath humid contents. This system is configured to detect the respiratory moisture flow that permeates from a mask wearer during breathing and coughing, to identify the signature of breath leakage associated with the form of face shielding. In the absence of face mask wearing during long exhale (Fig. 4F), a steep resistance increase (up to ~80%) is seen at the front fiber layer, followed by slower and weaker responses (up to ~20%) in the back and upper fiber layers (Fig. 4F_1_). In the case of cough, both the front and back fiber layers show obvious and similar resistance changes, suggesting that the airflow strength in cough is stronger than that of a long exhale (Fig. 4F_2_). In addition, the sound of the cough is captured by the middle piezoelectric P(VDF-TrFE) fiber array as seen in the inset graph. When wearing a surgical mask (Fig. 4G), the fiber resistance signal pattern during long exhale suggests that the surgical mask redirects some exhaled gas to leak out from the nose area and that the mask has greatly compromised the breath strength in the forward direction (Fig. 4G_1_). In the case of cough, the surgical mask does not seem to effectively prevent the moisture to pass through the mask, as indicated by the peaks in the front and back fiber layers (Fig. 4G_2_). When wearing an FFP2 grade (filters at least 94% of airborne particles) of respiratory mask (Fig. 4H), most of the moisture during a long exhale is detected by the upper sensor, possibly because of the gas leakage from the sealing between the mask and the nose area, instead of permeating through the mask fabric (Fig. 4H_1_). In addition, the respiratory mask is shown to be more effective to reduce the forward breath strength compared with the surgical mask during cough (Fig. 4H_2_). Both long exhale and cough in the above three situations have been repeated for continuous breathing, as seen in fig. S6 (C to G). Overall, with the material costs for these additively manufactured fiber sensing systems being around £0.5 for the single-layer sensor and around £2 for the trilayer 3D sensor, respectively, these portable, low-cost, disposal devices could see promising adaptation in assisting field mobile-health diagnostics and assessments (*40*).

### PEDOT:PSS fiber-based biointerface devices

**Fig. 5:**
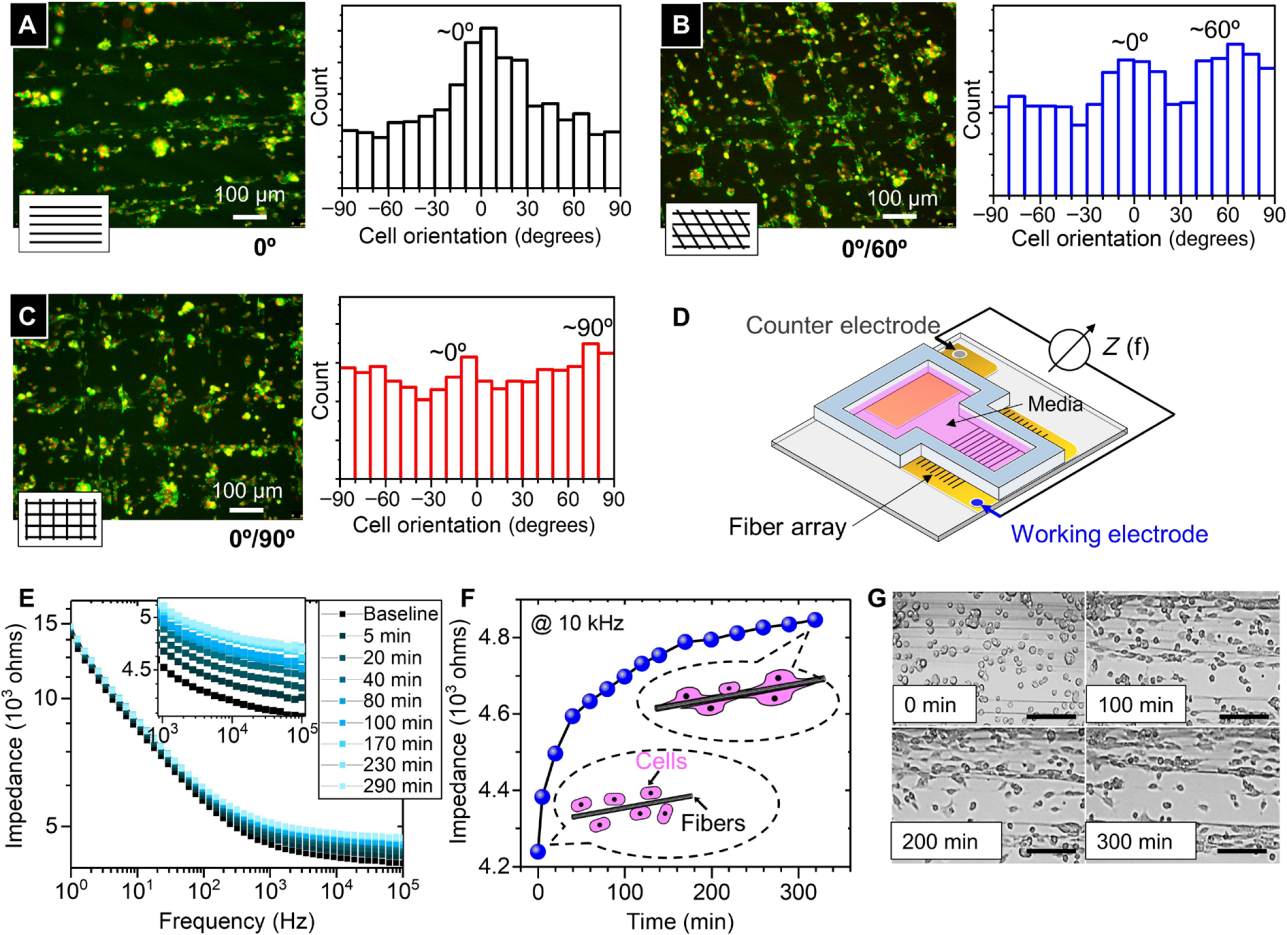
PEDOT:PSS fibers bioimpedimetric sensor. (**A** to **C**) Fluorescence images (red, nucleus; green, F-actin) showing fiber array guiding cellular orientation, as confirmed by the orientation histograms. (**D**) Schematic of the impedimetric sensor. (**E**) Time-lapsed in situ impedance measurement as a function of frequency. (**F**) Plot of impedance at 10 kHz versus time (after cell sedimentation), to compare with the time-lapsed imaging of cell-fiber interaction in (**G**). Scale bars, 150 μm.

PEDOT:PSS is known to have convenient cellular biocompatibility (*28*). Thus, we have used the fiber array to form biointerfaced sensing devices. We demonstrate that the PEDOT:PSS fiber array retains their designated patterns and orientation after liquid immersion and remains visually stable in cell culture media for at least 3 days (fig. S7, A to C). In other context, it was shown that PEDOT:PSS electrodes remain stable in cell culture media over a period of several months for in vitro electrophysiology applications (*41*). When PEDOT:PSS fibers are directly printed onto glass slides, upon cell seeding, the fibers are shown to facilitate spontaneous cell attachment and viable cell culture (viability over 3 days based on U87 cell test; fig. S7D). The percentage of cells that successfully attach to fibers is around 73% at day 3. The high aspect ratio of the fibers provides topographical guidance to the cells. As shown in Fig. 5 (A to C), 3T3 cells (fibroblast cells from mouse) follow the aligned fiber patterns over the entire fiber array. Different orientations of the fibers (0°/90°, 0°/60°, and 0°) guide the cells to align with respect to the fiber patterns, as highlighted in the associated histograms. To extend this capability further, we produce centimeter-long fibers (deposited on glass slides) to guide cellular alignment, where the fiber can simultaneously function as an impedimetric sensor detecting dynamics of cell coverage (Fig. 5D). Cell coverage on PEDOT:PSS electrode surfaces is known to increase the electrode’s sensing impedance (*42*). Coupled with simultaneous live-cell imaging permitted by the transparency of the fiber array, it is found that in our fiber-electrode setup, the impedance increases most noticeably during the initial stages of cell attachment and fiber-guided cell alignment (within approximatively 100 min from cell sedimentation). Figure 5E shows the impedance spectrum series of a typical time-lapse study, whereas Fig. 5F plots the impedance sampled at 10 kHz, of which evolution could be cross-correlated to the cellular dynamics data captured by time-lapse imaging in Fig. 5G and movie S3. In addition, preliminary experiments show that 3T3 cells could spontaneously attach to the fibers without any coating, forming elongated cell aggregates around the suspended fibers (fig. S7E). This could pave the way for future studies in 3D cell culture with in situ sensing.

### Creating circuit architectures using iFP fibers

The free-form nature of the iFP suspended conducting fiber arrays enables unconventional architectures harnessing its unique opto-electro-mechanical properties. As proof of concepts, we have fabricated 3D-layered “floating electronics” and suspended cross- (homo- and hetero-) junctioned fibers that cannot be easily achieved by existing fiber fabrication or printing techniques.

**Fig. 6:**
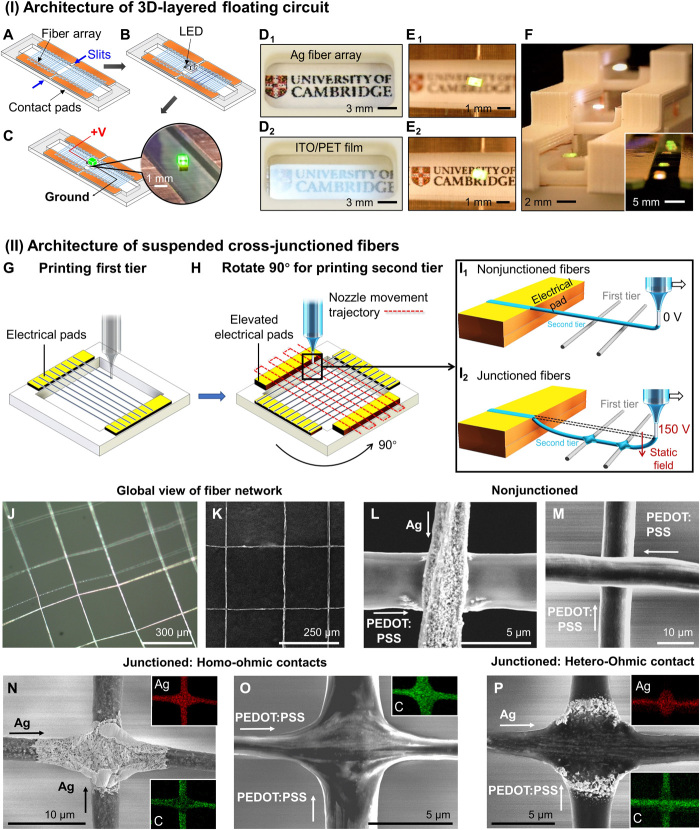
Creating circuit architectures using iFP fibers. (**I**) Architecture of 3D-layered floating circuit. (**A** to **C**) Schematics showing the successive steps to connect a LED on the suspended transparent Ag fiber array. (**D**_**1**_ and **D**_**2**_) Photos showing oblique light response from a suspended fiber array versus standard ITO/PET film. (**E**_**1**_ and **E**_**2**_) Top views of the floating circuit, in- and out of focus of the LED light. (**F**) Creating a 3D-layered circuit architecture by combining 3D printing, inset showing multiple-LEDs connection on a single array of PEDOT:PSS fibers. (Photo credit: Junyu Xie, University of Cambridge) (**II**) Architecture of suspended cross-junctioned fibers. (**G**) Schematic showing the printing of first fiber tier. (**H**) Schematic showing the frame is rotated 90° for printing the second fiber tier. (**I**_**1**_ and **I**_**2**_) Schematics showing the fabrication of nonjunctioned fiber networks (I_1_) and junctioned fiber networks assisted by an applied voltage (I_2_). (**J** and **K**) Optical and SEM global views of the suspended Ag and PEDOT:PSS junctioned fiber network. (**L** and **M**) SEM micrographs showing nonjunctioned fiber crossings. (**N** and **O**) SEM images showing homo-ohmic contacts of Ag or PEDOT:PSS fibers with the associated EDX mapping showing silver (Ag) and carbon (C) distribution at the junction, respectively. (**P**) SEM image showing the hetero-ohmic contact between Ag and PEDOT:PSS fibers with the associated EDX mappings.

For example, LED lamps can be directly powered through the transparent fiber electrode without soldering. Since the PEO sizer is only present as a thin sheath and has a low breakdown potential, the fiber array can be readily used as electrical conduits. Figure 6 (A to C) shows the simple successive steps to connect the LED circuit on the suspended Ag fiber array, where the LED lamp can be powered (~10 V). The fiber array is mechanically strong enough not only to support the weight of the LED lamp (1.4 mg) but also to bear the impact force when the light is placed on the fiber array manually with a tweezer. Since iFP can produce thin spanning fibers, the substrate-free conducting fiber arrays are noticeably more transparent by eliminating substrate absorption and minimizing the level of oblique light reflection compared with a standard transparent conductor film, i.e., ITO/PET with similar directional conductance (Fig. 6, D_1_ and D_2_). “Floating LED circuit” is created as demonstrated by the in- and out-focus images in Fig. 6 (E_1_ and E_2_). Furthermore, the sub-100°C fabrication process of iFP enables fiber array to be directly integrated onto materials with low melting temperatures, such as commonly used 3D printing plastics. This opens the way to low-cost and easily assembled 3D-layered “floating circuit,” as evidenced in Fig. 6F and fig. S8. Similarly, spanning PEDOT:PSS fiber array can also be used to connect and support multiple LED lamps on the same array (shown in the inset in Fig. 6F).

At the few micrometer scale, creating heterojunctions between organic and inorganic fibers is especially challenging when considering the very dissimilar processing conditions normally involved in manufacturing these fibers. However, the versatility fluidic printing feature of iFP enables materials of different nature to be in situ integrated into the same fiber network in a grid structure. In the production of a fiber network composed of orthogonal fiber arrays, the first tier of the fiber array is printed with the standard, zero-voltage iFP procedure (Fig. 6G). Afterward, the device is rotated 90°, for the printing of another fiber array in the transverse direction. This second-tier fiber array is spanned on contact pads at a raised level of ~30 μm compared to the first tier (Fig. 6H). In the case of a nonjunctioned fiber network, because the second fiber array tier is suspended at a different plane than the first fiber array tier, the fiber crossings are nonjunctioned and thus are insulated (Fig. 6I_1_). The first and second fiber tiers in the nonjunctioned network are electrically insulated because they are physically separated in different heights. In the case of a junctioned fiber network, the second fiber array tier is printed with applying a small voltage at the nozzle (~150 V with regard to the grounded translation stage). The static electric field (~75 V/m) would direct fibers in the second tier toward to the first fiber tier. As the semiliquid fibers from the second tier come into contact with the already deposited first tier, the residual solvent would dissolve the surface of the first fiber tier, thus creating fused fiber junctions (Fig. 6I_2_). The value of applied voltage is crucial to obtain fiber networks with uniform and controllable junctions. An applied voltage greater than 150 V would cause damage to the first fiber tier, while a lower voltage is insufficient to create well-fused junctions. The junctioned fiber networks are conducting, meaning that fibers between the first and second tiers can form ohmic contacts (fig. S9A and table S1B). Despite that the first tier fibers are subjected to substrate heating longer than the second tier fibers, control experiments in fig. S9B suggest that this does not change the conductivity and integrity of the fiber array.

A typical suspended, heterojunctioned fiber network of Ag and PEDOT:PSS fibers is shown by global optical and SEM images in Fig. 6 (J and K). Zoomed-in SEM images in Fig. 6 (L and M) show typical nonjunctioned fibers. SEM and EDX mappings in Fig. 6 (N and O) show the well-integrated homojunctioned (Ag-Ag or PEDOT:PSS-PEDOT-PSS) fibers, and heterojunctions between PEDOT:PSS and Ag fibers are shown in Fig. 6P. The merging at the cross-junctions happens when the second tier of semiliquid fibers comes to contact with the first tier of fibers, making this an in situ process working at sub-100°C. Thus, the iFP junctioning process differs from the existing methods, which reply on postthermal treatment or mechanical pressing (*43*). These postprocessing approaches could damage the fibers and, especially, cannot be applied for processing suspended fibers that are of a few micrometers in width. Thus, iFP is a unique and versatile method to create inorganic/organic cross-junctioned network without additional postprocessing. The ability of iFP to design fiber networks of different junction types, using different materials, may pave the way toward making transparent fiber-logic circuits (*44*).

## DISCUSSION

We have provided a first insight into a new way of producing on- and off-substrate fiber arrays using an original one-step approach, mitigating additional postprocessing that usually behoove to conventional fiber printing processes. iFP offers rapid and low-cost fabrication of high-performance transparent conducting fibers with minimized contact resistance and surface resistivity. The iFP fiber array can be directly coupled with materials of low operation temperatures (<100°C), starting from solutions or dispersions of low-viscosity fluids.

We have demonstrated the universality of iFP in producing PEO-sheathed fibers made of metal (Ag) and conducting polymer (PEDOT:PSS), respectively. Both Ag and PEDOT:PSS fibers exhibit good bond-to-bond fiber conductivities. By readily fabricating an in-plane array of fibers and assembling them into 3D architectures, iFP multiplies the conduction ability of single fibers while still harnessing the superior optical transparency through single-fiber thicknesses. With this capability, all the fiber sensor structures presented in this work have supported convenient imaging acquisition, which is important for data validation, and also enabling future potentials in multiparametric studies of process dynamics. When constructed as a moisture sensor, suspended PEDOT:PSS fiber array demonstrates superior responsiveness compared to the conventional film-based sensing devices originated from the fibers’ small unit volume and high surface area–to–volume ratio. This enables the fiber arrays to effectively detect high-frequency breathing events. Further, a layer-by-layer 3D fiber array permits the spatiotemporal flow dynamics to be captured, which is not possible with existing film-based sensors. These transparent and permissive multilayer fiber arrays demonstrate unique and integrated sensing capability in low-cost and wearable/portable breath analysis applications. The versatile and facile device integration also inspires new concepts in circuitry architectures. Using the superior optical transparency and minimal reflection of the substrate-free fibers, we demonstrated 3D-layered floating electronics. In addition, we constructed suspended fiber networks of homo- or heterofiber cross-junctions. Thus, by expanding the material combinations in the future, the iFP process is foreseen to offer a tool to fabricate conducting fibers with a tunable combination of mechanical, optical, and electrical properties, broadening the property scopes offered by existing transparent conducting fiber and network structures. The iFP process has brought new functional dimensions to the small-diameter conducting fiber structures. We envisage these distinctive forms of transparent fiber array and networks to be exploited in emerging fields from bioelectronics and 3D electronics to Mobile Health.

## MATERIALS AND METHODS

### Solution preparation

The reactive Ag ink was prepared with modified Tollens’ ink (*29*): 1 g of Ag acetate (Sigma-Aldrich) was dissolved in 2.5 ml of ammonium hydroxide (28% NH_3_, Alfa Aesar), and then 0.2 ml of formic acid (Thermo Fisher Scientific) was added, followed by 3-hour stirring at room temperature to obtain a transparent solution. PEDOT:PSS solution was prepared by mixing 95% (v/v) of PEDOT:PSS (Heraeus Clevios PH 1000) and 5% (v/v) of ethylene glycol (Sigma-Aldrich). The solution was sonicated for around 20 min and then filtered through a 1.2-μm PTFE (polytetrafluoroethylene) filter before using. To enhance the structure and conductivity stability of PEDOT:PSS fibers in the case of using them inside cell culture media, 1% (w/w) of DVS (divinylsulfone; Sigma-Aldrich) was then added to the solution as a cross-linker. DVS is able to cross-link PEDOT:PSS under room temperature, and the cross-linked PEDOT:PSS is proven to be biocompatible. After mixing with DVS, the solution was mixed carefully and filtered through a 1.2-μm PTFE filter. PEO (*M*_w_ = 4 million Da) was dissolved in DI water at 2% (w/w) and stirred at room temperature for 24 hours.

### iFP of fiber array

The iFP setup consisted of two X-*Y* translation stages (DDSM100/M, Thorlabs) for fiber printing and a *Z* stage (L490MZ/M, Thorlabs) to adjust the distance between the nozzle and the contact pad. The core-shell nozzle was assembled by inserting a micropipette-filling capillary (inner diameter of 100 μm and outer diameter of 164 μm; World Precision Instruments) into a 22-gauge blunt-end stainless steel needle. The nozzle was kept at a fixed position. Two pumps (AL-1000, World Precision Instruments) were used to deliver the PEO sizing solution and functional core solutions into the sheath and core channels of the core-shell nozzle, respectively, via 0.8-mm inner-diameter tubings. In the fabrication of the fiber-based sensors and devices, the iFP sheath solution flow rate was maintained at ~50 μl/hour for the injection of the 2% (w/w) 4 million Da PEO sizing solution, and the core flow rate was maintained at ~25 μl/hour for the injection of functional solutions. A heating plate was attached to the bottom of the translation stage and was continuously heated at 80°C for Ag fiber production and 65°C for PEDOT:PSS fiber production to assist reduction reaction and solvent evaporation, respectively. During the fiber printing process, the translation stage moved at a speed of 600 mm/s, and the distance between two electrical contact pads, which is fiber spanning length, could be varied between 1 and 10 mm. In the process of printing fibers bond to the surface of a substrate, a small voltage (~150 V) was applied on the tip of the nozzle to pull the printed fibers down to adhere to the substrates. The distance between the tip of the nozzle and the translation stage substrate was maintained at ~5 mm; thus, the fibers arrays are printed to be suspended at ~5-mm height above the translation stage.

### Fiber characterization

SEM micrographs were acquired with FEI Nova NanoSEM (accelerator voltage, 5 kV), and the EDX mappings were taken with a silicon Drift Detector EDX spectrometer fitted with the SEM (accelerator voltage, 10 kV). Prior coating was not needed because the fibers were conducting. For both suspended Ag and PEDOT:PSS fibers fabricated with 2% (w/w) 4 million Da PEO sheath, under core/shell flow rates of 25 and 50 μl/hour, their diameters were measured from analyzing more than 50 fibers (in each case) from five different samples in randomly selected field of views under SEM. It is shown that the average suspended fiber diameter is 2 μm with an SD of 0.7 μm. When deposited onto substrates, the Ag fiber track width is maintained at 2 ± 1 μm. TEM micrographs were acquired with FEI Tecnai F20 with 200-kV field emission gun high-resolution and analytical TEM (accelerator voltage, 80 kV). XPS spectra of the Ag fiber bond were collected by ESCALAB 250Xi photoelectron spectroscopy with monochromatic Al Kα excitation (1486.68 eV). A depth profile was conducted to acquire information of different depth layer (Ar + ion gun, 2000 eV), with the etching time of 350 s for each etching layer. The Ag fiber bond was printed onto the copper contact pad made of copper tape, and then the copper tape was directly adhered to the XPS sample holder. Before acquiring the XPS spectrum at layer 0, the ion gun had already been operated for dozens of seconds to remove the environmental carbon contaminations. The high-resolution spectroscopies of Ag 3d and C 1s were acquired in each etching layer, and the Ag and C elements were determined with relative sensitivity factors (from the AlKalpha Scofield library) of 10.66 and 1.0, respectively. In addition, XPS survey was also acquired in each layer, and the Cu 2p peak was determined from the XPS survey with relative sensitivity factor of 25.39. The analysis area was around 100 μm. The data were extracted from the XPS machine and analyzed with Thermo Fisher Scientific Avantage software. UV-visible spectra were collected by the Cary 7000 Universal Measurement Spectrophotometer (Agilent Technologies) with suspended fiber array samples, and the fiber density used for the measurement was 20 fibers/mm. The XRD measurement for silver fibers was carried out by Siemens D500 (SIEMENS) with the 35-kV and 20-mA x-ray setting. The crystallinity was calculated from the area under crystalline peaks over total scattering area, and the average crystalline grain size was determined with the Scherrer equation (*45*). During the XRD measurement, silver fibers and silver ink were deposited onto separate microscope cover slides, which were mounted onto the XRD sample holder directly. The scanning step size was 0.02°. TGA measurement was performed by the TA Instruments Q500 TGA machine, with a heating rate of 10°C/min under nitrogen environment. The stress-strain behavior of fibers was measured on samples with an array of fibers (around 100 fibers per sample). Since suspended fibers are substrate free and extremely thin, the conventional tensile test machine was not suitable. A self-assembled tensile rig was used as illustrated in Fig. 2B. Fibers were pulled up in the middle by a probe, which was connected to a force sensor. The pulling force and displacement were recorded and later converted into stress and strain of the fiber according to the following equation

$$\text{Strain}=\frac{2\sqrt{D^{2}+{\left(L/2\right)}^{2}}-L}{L}\times 100\%;\mathrm{stress}=\frac{F}{2\cdot \text{cos}(\alpha /2)\cdot A}$$

where *D* is the displacement of the probe, *L* is the original fiber length, *F* is the force measured by the probe, α is the angle of the fiber at the tip of the probe, and *A* is the original cross-sectional area of the fiber.

### Fabrication of moisture flow and respiratory sensors

In the PEDOT:PSS moisture sensor, a commercial humidifier was used as the mist source, and multimeters (Keysight 34465A) were used to record the resistance change. High-speed camera (Phantom VEO-E 310 L) was used to image the moisture flow. In fabricating the single-layer fiber wearable respiratory sensor, suspended PEDOT:PSS fibers were printed onto a 3D-printed frame (acrylonitrile butadiene styrene; ABS materials, 10 mm by 8 mm by 2 mm), with the contact pads made of copper tape (33 μm in thickness; RS Components). The fiber sensor was directly attached on the exterior of a disposable face mask. The resistance of the fiber was recorded by a Keysight 34465A multimeter. In the portable trilayer fiber sensor, the suspended PEDOT:PSS and P(VDF-TrFE) fibers were printed onto the same 3D-printed frames and were stacked together. The sensor was directly attached onto the front camera of a cell phone (iPhone 11 Pro). In this case, the resistance of all PEDOT:PSS fiber arrays were monitored by a single Arduino UNO board, and the voltage output from the P(VDF-TrFE) piezoelectric fibers was collected by an oscilloscope (Tektronix TDS 2014). The sterile surgical mask used in experiments was fitted with YY0469 standard, and the FFP2 respirator mask used was fitted with EN149-2009 standard.

### Cell viability assay and fabrication of cell-interfacing devices with PEDOT:PSS fibers

Human glioblastoma cells, U-87 (American Type Culture Collection), were cultured in Dulbecco’s modified Eagle’s medium (DMEM, Invitrogen) supplemented with 10% (v/v) fetal bovine serum (FBS; Sigma-Aldrich) under 5% CO_2_ at 37°C incubation. Cells were seeded from a centrifuged cell pellet directly on fibers. For the viability study, a seeding volume of 10 μl of cell pellet was used for each sample; samples were cultured for 3 days and imaged with optical microscopy. A live/dead assay (Invitrogen) was performed following the manufacturer’s protocol on day 3 and imaged with a Leica SP5 confocal microscope. The cell attachment percentage was calculated from three independent samples with fibroblast 3T3 cells on PEDOT:PSS fibers. In each sample, three randomly chosen fields of view on each sample were studied. For fabricating cell-interfacing devices, PEDOT:PSS fibers of 20 mm in length were printed onto glass slides with a patterned gold electrode (CRESTEC CABL 9000C E-Beam Lithography System). All samples were kept in room temperature for 24 hours before immersing into cell culture media, to make sure the completion of cross-linking process. The device was then sterilized under UV radiation for 30 min. Afterward, the device was washed three times by phosphate-buffered saline and incubated in cell culture media [85% (v/v) DMEM, 10% (v/v) FBS, 5% (v/v) penicillin-streptomycin; Thermo Fisher Scientific] for 48 hours to remove excessive PSS in the PEDOT:PSS fibers, and the media was refreshed before cell seeding. This conditioning process also ensured the impedance of the PEDOT:PSS fibers to stabilize in the aqueous environment. As shown in the control experiment in fig. S7C, the impedance of PEDOT:PSS fibers fluctuated and increased in the first 30 hours of soaking, possibly because of water uptake and nonspecific interaction between the fiber and the molecules present in the culture media. Afterward, the fiber impedance remained stable throughout. Thus, the cell-fiber interaction experiments in Fig. 5 would be due to the net effects of the presence of cells. Fibroblast cells (3T3) were seeded on the device, with the impedance spectrum sampled by PalmSens4 Potentiostat along with in situ imaging (Leica SP5 confocal microscope) under 5% CO_2_ at 37°C incubation. Fluorescent images in Fig. 4 (A to C) with phalloidin and 4′,6-diamidino-2-phenylindole stains were taken by fixing cells at 8 hours, and cell orientations were analyzed using CellProfiler 2.3.

## SUPPLEMENTARY MATERIALS

Supplementary material for this article is available at http://advances.sciencemag.org/cgi/content/full/6/40/eaba0931/DC1
